# Behavior of Residual Current Devices at Earth Fault Currents with DC Component

**DOI:** 10.3390/s22218382

**Published:** 2022-11-01

**Authors:** Stanislaw Czapp, Hanan Tariq, Slawomir Cieslik

**Affiliations:** 1 Faculty of Electrical and Control Engineering, Gdańsk University of Technology, Narutowicza 11/12, 80-233 Gdańsk, Poland; 2 Faculty of Telecommunications, Computer Science and Electrical Engineering, Bydgoszcz University of Science and Technology, 85-796 Bydgoszcz, Poland

**Keywords:** DC component, residual current devices, sensitivity, sensors testing and evaluation

## Abstract

Low-voltage electrical installations are increasingly saturated with power electronic converters. Due to very high popularity of photovoltaic (PV) installations and the spread of electric vehicles (EV) as well as their charging installations, DC–AC and AC–DC converters are often found in power systems. The transformerless coupling of AC and DC systems via power electronic converters means that an electrical installation containing both these systems should be recognized from the point of view of earth fault current waveform shapes. In such installations, various shapes of the earth fault current may occur—a DC component of a high value may especially flow. The DC component included in the earth fault current influences the tripping threshold of residual current devices (RCDs)—the devices which are mandatory in certain locations. This paper presents results of the AC-type, A-type, and F-type RCDs sensitivity testing under residual currents of various compositions of the DC component. This testing has shown that the DC component may both degrade and improve the sensitivity of RCDs. Moreover, unexpected positive behaviors of RCDs in some circumstances under DC residual current is discussed. Therefore, recognizing the real sensitivity and behavior of RCDs from the point of view of the DC component is important for effective protection against electric shock, in particular, in PV installations and EV charging systems. The research results provide a new insight into the real behavior of RCDs in modern power systems and, consequently, the safety of people.

## 1. Introduction

Air pollution coming from the classic way of power production as well as from utilization of combustion engines in transportation systems has nowadays resulted in a rapid development of photovoltaic (PV) power generation and an increased number of electric vehicles (EV). PV systems and EV charging systems include installations composed of alternating current (AC) circuits and direct current (DC) circuits. To utilize power generated in PV systems, the use of the DC–AC power electronic converters is necessary. These converters (inverters) may have various topologies [1,2,3] and their topologies along with earthing systems [4] influence the earth–current phenomena occurring during the normal operation as well as in the case of the earth fault. In transformerless PV installations, their components influence the leakage current’s flow [5,6], and for functional purposes, in some cases, these currents are required to be strongly reduced [7,8] with the use of advanced types of inverters [9] or even a unique pulse width modulation method [10]. Values of the leakage current may also depend on the type and mounting conditions of PV modules [11]. In turn, faults in PV systems [12,13], especially earth faults [14] may introduce a threat to the safety of people and equipment. It mainly refers to the risk of an electric shock [15] as well as the risk of fire [16], arising especially during series arcing [17,18]. Similar types of risk occur in EV charging installations [19,20] that require the use of advanced methods of EV connection with the power system [21,22,23].

**Figure 1:**
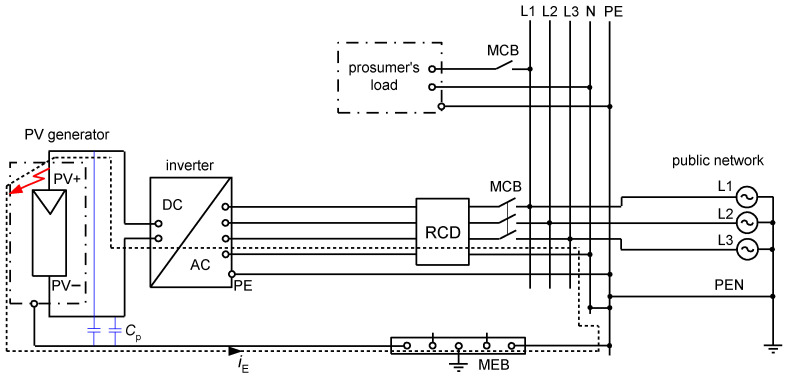
An example installation with photovoltaic (PV) sources of energy and earth fault current *i*_E_ flow in the case of the earth fault at the DC side. RCD—residual current device, MCB—circuit-breaker, MEB—main equipotential bonding busbar, *C*_p_—capacitance-to-earth in a PV installation.

**Figure 2:**
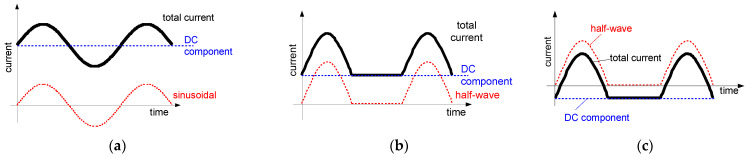
Composite waveforms of earth fault currents (“total current”) in the coupled DC–AC systems. Components: (**a**) sinusoidal AC + smooth DC; (**b**) pulsating half-wave + smooth DC of same polarity; (**c**) pulsating half-wave + smooth DC of opposite polarity.

Taking properties of PV installations and EV charging installations into account, one can conclude that evaluation of the effectiveness of electrical safety, i.e., protection against fire and especially protection against electric shock in case of an insulation fault in such installations is not an easy task. Deep analysis of phenomena occurring during earth faults is very important especially when residual current devices (RCDs) are applied. Electrical installations composed of both DC and AC systems which are coupled via transformerless converters (inverters), as well as installations having other types of converters, e.g., variable-speed drive systems, may generate the earth fault currents of various waveform shapes. Figure 1 presents an example installation with PV sources of energy and the earth fault current path in the case of an earth fault at the DC side of the installation. As concluded in [24], the resultant earth fault current in such an installation is composed of the AC component and the DC component and the waveform shape depends on the inverter type and its topology. Example composite waveforms are presented in Figure 2.

Problems with protection against electric shock, including residual current protection in circuits with power electronic converters refer mainly, in the literature, to high-frequency components of the earth fault current and their negative effect on the tripping of RCDs. With reference to high frequency currents, this negative effect is studied in [25,26,27], whereas with reference to earth fault currents containing harmonics in [28,29]. The results of the investigations have shown that RCDs may have difficulties with detection of earth fault currents composed of harmonics. Due to high frequency components, RCDs may have a significantly higher tripping threshold or even not react at all. It is dangerous in terms of the effectiveness of protection against electric shock. A similar conclusion is included in paper [30] which considers EV charging systems. Negative effect of converters on RCDs also occurs during normal operation of installations—as presented in [31], RCDs may be a cause of the unwanted disconnection of PV systems due to high-value leakage currents.

**Figure 3:**
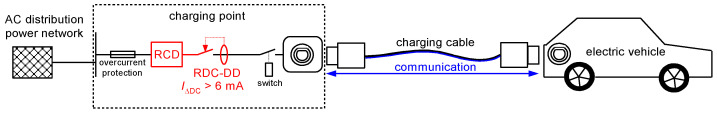
An example supply system for charging of electric vehicles. RCD—residual current device, RDC-DD—residual direct current detecting device.

A review of the literature has shown that in the case of installations containing direct current sources, very little attention is paid to the sensitivity of RCDs and their tripping testing under direct currents, although there are more and more PV and charging EV installations having DC circuits. Moreover, there is a fairly common opinion that the earth fault current with a large share of the DC component can be detected only by the most technically advanced and the most expensive B-type RCDs. Some results for B-type RCDs are discussed in [32]. The simulative study presented in [24] shows serious theoretical problems of the RCDs operation in PV systems in the case of the earth fault at the DC side of the installation. The waveform shapes may be unidirectional, unconventional and, hence, theoretically very difficult to be detected by simpler RCDs—i.e., A-type RCDs, the most widespread in various low-voltage installations. The problem of the insufficient ability of A-type RCDs for the detection of DC currents is also highlighted in the standard [33]. If high value of the DC component is expected (higher than 6 mA), the A-type RCD should be accompanied by an additional device—a residual direct current detecting device (RDC-DD in Figure 3), to ensure effective detection of the earth fault current.

However, the authors of this paper conducted a wide testing of A-type and F-type RCDs under earth fault currents of high DC component and results of tests have shown that in the condition of a real earth fault these types of RCDs may react unexpectedly positive. This gives a new perspective and a new evaluation of the possible sensitivity and behavior of RCDs under the earth fault/residual current having a high DC component, in particular with regard to the state of the art contained in international standards. The research results and conclusions contained in this paper are very important for the safety of people. The results of the analyses and tests obtained by the authors are also a guideline for manufacturers/constructors of RCDs dedicated to circuits in which a high DC component may appear in the earth fault current.

The rest of the paper is organized as follows. Section 2 presents classification of RCDs as well as earth fault current waveform shapes designated for RCDs sensitivity testing. The construction of the laboratory setup for RCDs testing along with results of the tests and related discussion are included in Section 3. Final conclusions, including proposals for modification of RCDs classifications as well as recommendations for manufacturers of RCDs, are presented in Section 4.

## 2. RCD Classification and the Scope of RCDs Testing

**Figure 4:**
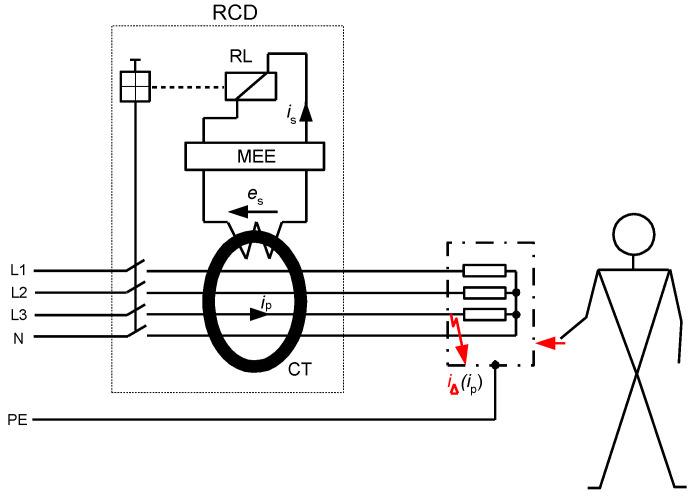
Simplified structure of the residual current device (RCD). RL—relay, MEE—matching electronic elements (optional), CT—current transformer (current sensor), *e*_s_—induced secondary voltage in CT, *i*_p_—primary current of the CT, *i*_s_—secondary current of the CT, *i*_∆_—earth fault (residual) current.

An RCD is a protective device, the use of which is recommended for protection against electric shock, and in some installations its use is even mandatory. The mandatory use of RCDs, especially those of a rated residual operating current equal to 30 mA, is specified in standards, e.g., in [34,35]. However, the selection of RCDs for given circuits has to be conducted very carefully because their protective properties strongly depend on the type of RCD vs. expected waveform shape of the earth fault current. To ensure tripping of the RCD in the case of earth fault, the secondary current *i*_s_ of enough high value has to flow through relay RL (Figure 4). The value of the current *i*_s_ depends on the value of the induced voltage *e*_s_ in the secondary winding of the current transformer/sensor CT. In turn, the value of this voltage depends on the primary current *i*_p_ (earth fault/residual current *i*_∆_) and properties of the iron core of the current transformer CT. Therefore, it is evident that for RCDs containing an iron core transformer (typical solution), the main problem is the transformation of the unidirectional currents. To increase the ability of RCDs to detect such currents or ensure the delay in their tripping, secondary circuit of the RCD may include additional matching electronic elements (MEE).

From the point of view of the ability to detect various shapes of the earth fault/residual currents, RCDs are divided into four groups (according to [36,37]), as shown in Table 1. This table also includes example normative waveform shapes containing the DC component or direct current of low pulsation, under which particular types of the RCDs are tested.

As shown in Table 1, AC-type RCDs are not designed to detect any types of DC waveforms but in many countries, they are used almost exclusively (low cost), whereas B-type RCDs are able to detect even smooth direct currents and, therefore, they may be applied in EV charging installations [33] as well as in PV installations [38] without limit. This is the effect of the B-type RCD’s special construction but, unfortunately, it requires an auxiliary voltage for detection of direct currents. Another disadvantage of B-type RCDs is the very high cost. For these reasons, the possibilities of using RCDs of a different type (A-type, F-type) are analyzed in each case during the project stage.

Taking the aforementioned into account, AC-type, A-type, and F-type RCDs are interesting to verify their real behavior under DC waveforms (wider than normative). Therefore, the following tests of the RCDs have been performed:

(1) TEST 1: verification of the tripping level of RCDs for slowly rising AC sinusoidal waveform in presence of a smooth DC component;
(2) TEST 2: verification of the tripping level of RCDs for slowly rising pulsating (half-wave) component in presence of a smooth DC component;
(3) TEST 3: verification of the tripping level of RCDs in the case of the suddenly applied pure direct current of the predetermined value. This type of test reflects a real accident of the electric shock and the most probable behavior of RCDs. If there is an earth fault (damage to insulation of the current-using equipment), the current suddenly starts flowing towards the earth. It is similar when a person touches an enclosure of the faulty current-using equipment—the body current appears suddenly.

The aim of the above-described tests is to find out the real ability of A-type, F-type, and AC-type RCDs for detection of earth fault currents containing a high-value DC component as well as the RCD’s response to the pure direct current which is suddenly applied. Determination and evaluation of the real behavior of such types of RCDs would enable their broader use instead of the most expensive B-type RCDs.

## 3. Testing of RCDs

### 3.1. General Information

**Figure 5:**
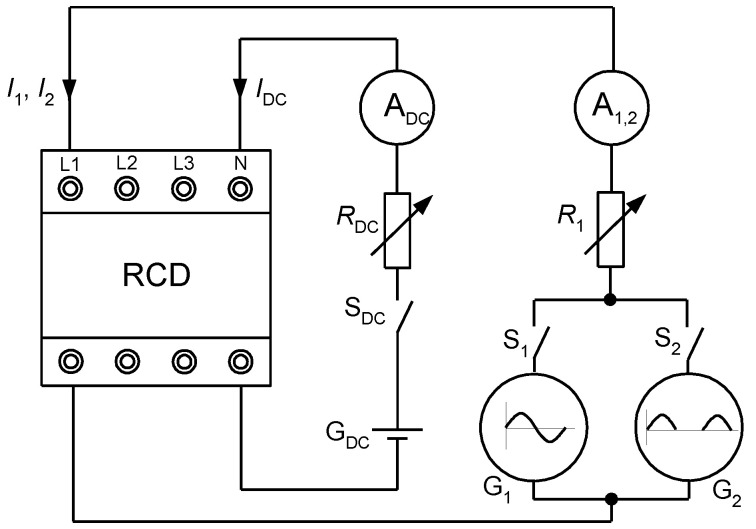
Simplified structure of the laboratory setup for RCD testing under residual currents with DC component or pure direct currents. A_DC_—ammeter for DC currents; A_1,2_—ammeter for AC currents and pulsating DC currents; G_DC_—generator for producing smooth DC component; G_1_—generator for producing AC sinusoidal component; G_2_—generator for producing pulsating DC component; *R*_DC_, *R*_1_—rheostats for limiting values of the testing current; S_DC_, S_1_, S_2_—switches; *I*_DC_—smooth DC component; *I*_1_—AC sinusoidal component; *I*_2_—pulsating DC component; RCD—tested residual current device.

The testing of RCDs has been performed with the use of the laboratory setup of the structure presented in Figure 5. During TEST 1, after closing switch S_DC_, the generator G_DC_ and rheostat *R*_DC_ are used for setting the proper value of the DC component (*I*_DC_ = 6, 15, 30, 60 or 150 mA). When predetermined DC component *I*_DC_ is forced, then the switch S_1_ is closed and the generator G_1_ forces the sinusoidal waveform *I*_1_, whose value is increased until the RCD trips. This way of forcing the waveforms enables mutual superimposition of the pure DC component and AC sinusoidal component. In this test, the rms value of the AC sinusoidal component, which initiates tripping of the RCD, is measured.

In the case of TEST 2, a similar principle applies. The difference is that instead of generator G_1_, generator G_2_ is used, and a pulsating direct current (half-wave) is forced. In this test, the rms value of the pulsating current is measured.

TEST 3 is performed on a different principle. Here, only the generator G_DC_ is used. After the circuit parameters are set to force a prospective smooth DC current of one of the following values: 15, 20, 30, 60, 90, 150, 300, or 600 mA, the switch S_DC_ is closed, which means there is a sudden DC current flow (it reflects a sudden contact of person with enclosure under dangerous voltage). During this test, the tripping/no tripping of the RCD is recorded.

### 3.2. Testing under Composite Current with DC Component

For the testing under composite currents containing a smooth DC component, the following RCDs have been specified:

- three A-type RCDs of a rated residual operating current *I*_∆n_ = 30 mA; these RCDs are marked RCD30_1, RCD30_2, and RCD30_3;
- two F-type RCDs of a rated residual operating current *I*_∆n_ = 30 mA; these RCDs are marked RCD30_4, RCD30_5;
- two A-type RCDs of a rated residual operating current *I*_∆n_ = 100 mA; these RCDs are marked RCD100_1, RCD100_2;
- two A-type RCDs of a rated residual operating current *I*_∆n_ = 300 mA; these RCDs are marked RCD300_1, RCD300_2;
- two AC-type RCDs of a rated residual operating current *I*_∆n_ = 30 mA; these RCDs are marked RCD30_AC1, RCD30_AC2.

**Figure 6:**
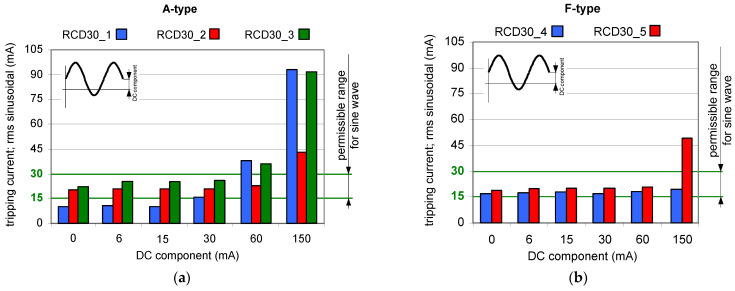
Tripping current (rms value of the AC sinusoidal component) of 30 mA RCDs under AC sinusoidal current superimposed by smooth DC component of values 6, 15, 30, 60, and 150 mA: (**a**) A-type RCDs (three tested RCDs); (**b**) F-type RCDs (two tested RCDs).

**Figure 7:**
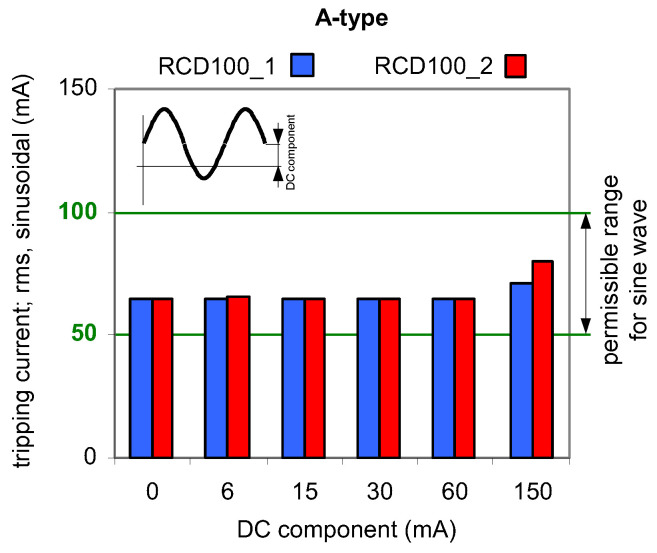
Tripping current (rms value of the AC sinusoidal component) of two 100 mA A-type RCDs under AC sinusoidal current superimposed by smooth DC component of values 6, 15, 30, 60, and 150 mA.

**Figure 8:**
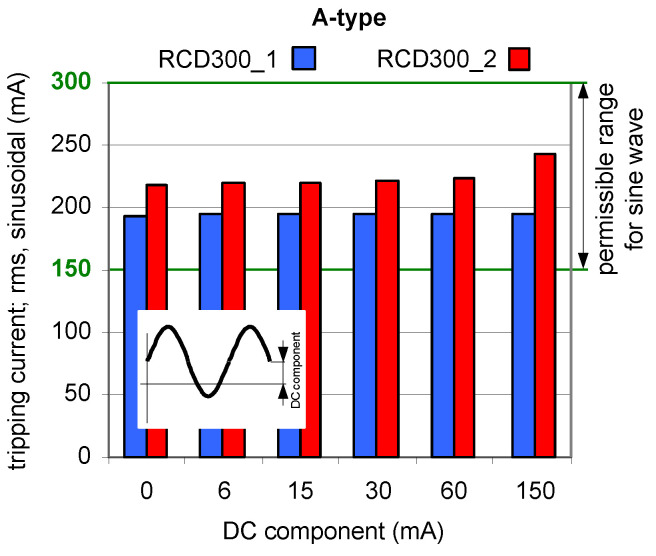
Tripping current (rms value of the AC sinusoidal component) of two 300 mA A-type RCDs under AC sinusoidal current superimposed by smooth DC component of values 6, 15, 30, 60, and 150 mA.

**Figure 9:**
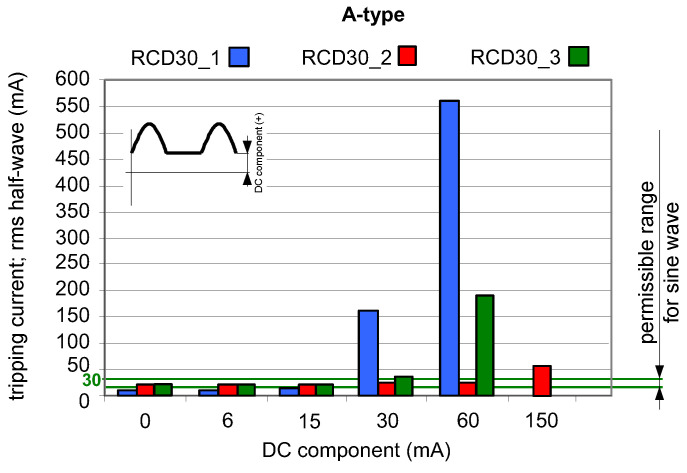
Tripping current (rms value of the DC pulsating component) of three 30 mA A-type RCDs under pulsating DC (half-wave) current superimposed by smooth DC component of values 6, 15, 30, 60, and 150 mA. Both components have positive (+) polarity.

**Figure 10:**
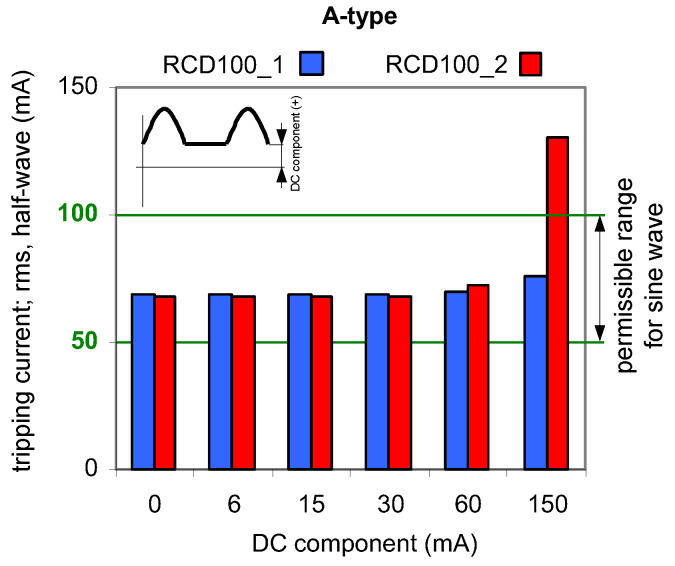
Tripping current (rms value of the DC pulsating component) of two 100 mA A-type RCDs under pulsating DC (half-wave) current superimposed by smooth DC component of values 6, 15, 30, 60, and 150 mA. Both components have positive (+) polarity.

**Figure 11:**
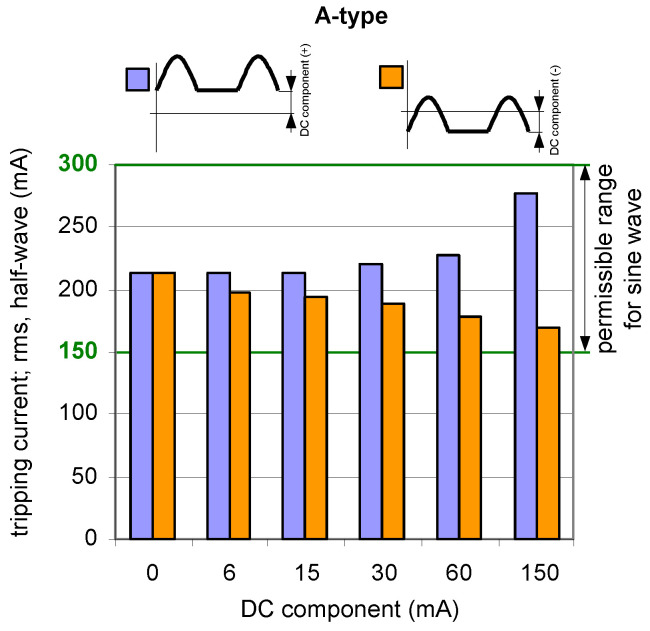
Tripping current (rms value of the DC pulsating component) of a 300 mA A-type RCD (symbol RCD300_2) under pulsating DC (half-wave) current superimposed by smooth DC component of values 6, 15, 30, 60, and 150 mA. Note—the smooth DC component may have a positive (+) polarity (left-side bars) or a negative (−) polarity (right-side bars).

Results of TEST 1 are presented in Figure 6, Figure 7 and Figure 8 whereas of TEST 2 in Figure 9, Figure 10 and Figure 11. In each figure with the results, the normative permissible tripping range for a pure sinusoidal current is marked (0.5–1.0)*I*_∆n_, i.e., for a 30 mA RCD it is 15–30 mA (green values on vertical axis). For considerations conducted in the paper this range is assumed as a proper one and a reference.

The testing of 30 mA A-type RCDs under a composite current having a sinusoidal AC and a smooth DC has shown (Figure 6a) that the higher the value of the latter component, the higher the tripping threshold of the RCDs. However, it is seen that even for the DC component equal to 30 mA, the rms values of the AC sinusoidal component which initiate tripping of RCDs are not higher than *I*_∆n_ = 30 mA (upper assumed limit). The most favorable behavior refers to RCD30_2 for which the tripping threshold is within the normative AC range when the DC component is equal up to 60 mA. This RCD has the best behavior for the 150 mA DC component as well.

F-type 30 mA RCDs (Figure 6b) have better immunity to the DC component (compared to A-type 30 mA). For device RCD30_4, the tripping rms value of the AC sinusoidal component is below *I*_∆n_ = 30 mA even when the DC component is five times higher, i.e., 150 mA.

The results obtained for 100 mA RCDs (Figure 7) and 300 mA RCDs (Figure 8) show that for all values of the DC component the rms values of the AC sinusoidal component which is responsible to initiate tripping of RCDs are within (0.5–1.0)*I*_∆n_. This behavior of the RCDs is very favorable. The comparison of the results for 30 mA vs. 100 mA, and vs. 300 mA RCDs shows that the ratio of DC component/rated residual operating current *I*_∆n_ is important. The RCDs with a greater value of *I*_∆n_ will be relatively more resistant to a DC component of a given value.

The analysis of the results obtained during TEST 2 (the A-type RCDs and AC-type RCDs have been tested) allows us to present conclusions that the composition of the pulsating (half-wave) component and smooth DC component may give unexpected behavior of RCDs—Figure 9, Figure 10 and Figure 11. The key element is the direction (polarity) of each component. If the two above-mentioned components have the same direction (polarity), the increase of the smooth DC component makes the composite current less pulsating and the effect of the smooth DC component is unfavorable—the tripping current increases (see results in Figure 9 and Figure 10).

If the polarities of the half-wave component and the smooth DC component are opposite, the positive behavior of RCDs may be met. Paradoxically, the smooth DC component may improve sensitivity of the RCD then. It is clearly seen in Figure 11 where tripping of the 300 mA RCD is shown for the following two cases:

(1) the half-wave component is positive (+) and the smooth DC component is positive (+) as well;
(2) the half-wave component is positive (+) but the smooth DC component is negative (−).

In case (1), the tripping threshold rises when the smooth DC component is being increased. The opposite phenomenon is recorded for the aforementioned case (2)—the higher the DC component, the lower the tripping threshold is. This is because increasing the smooth DC component to some extent makes the resultant/composite waveform bidirectional (see example waveforms in Figure 2c). It favors detection of the residual current, and, therefore, the sensitivity and tripping of the RCD.

**Figure 12:**
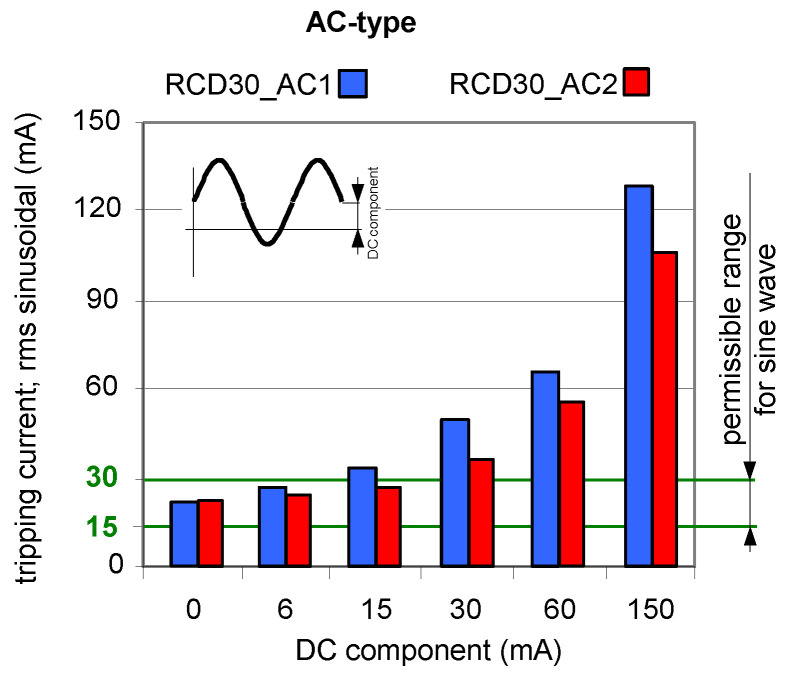
Tripping current (rms value of the AC sinusoidal component) of two 30 mA AC-type RCDs under AC sinusoidal current superimposed by smooth DC component of values 6, 15, 30, 60, and 150 mA.

**Figure 13:**
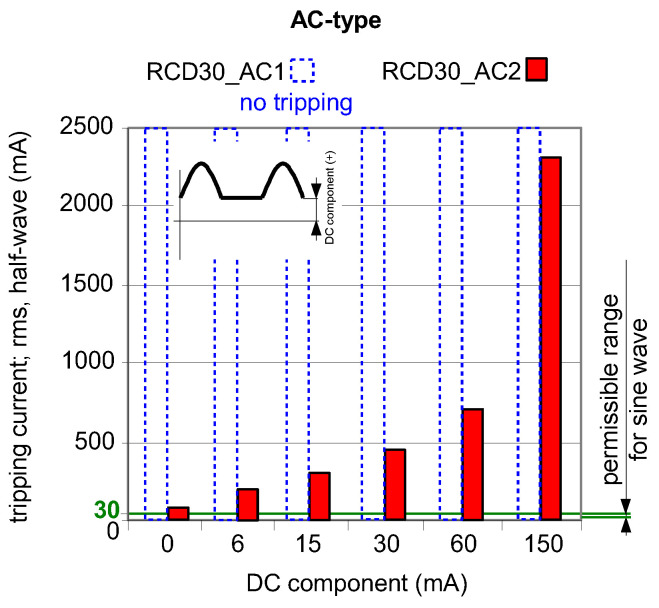
Tripping current (rms value of the DC pulsating component) of two 30 mA AC-type RCDs under pulsating DC (half-wave) current superimposed by smooth DC component of values 6, 15, 30, 60, and 150 mA. Both components have positive (+) polarity.

As aforementioned in Section 2 of this paper, AC-type RCDs are almost exclusively used in many countries due to their low cost. This is risky as there are more and more installations where a DC component may occur in the event of an earth fault. To prove this, Figure 12 and Figure 13 show the test results of two AC-type RCDs (symbols: RCD30_AC1 and RCD30_AC2). For the current waveforms consisting of AC component and smooth DC components, both RCDs still reacted (Figure 12). Unfortunately, for the current waveforms consisting of half-wave and a smooth DC component (Figure 13), the RCD30_AC1 did not respond to the test current at all. Moreover, with the DC component equal to 150 mA, the second AC-type RCD of *I*_∆n_ = 30 mA (RCD30_AC2) had a highest tripping current of as much as 2.3 A. This is unacceptable from the point of view of protection against electric shock and fire.

### 3.3. Modeling the Operation of RCDs in the Presence of the DC Component

**Figure 14:**
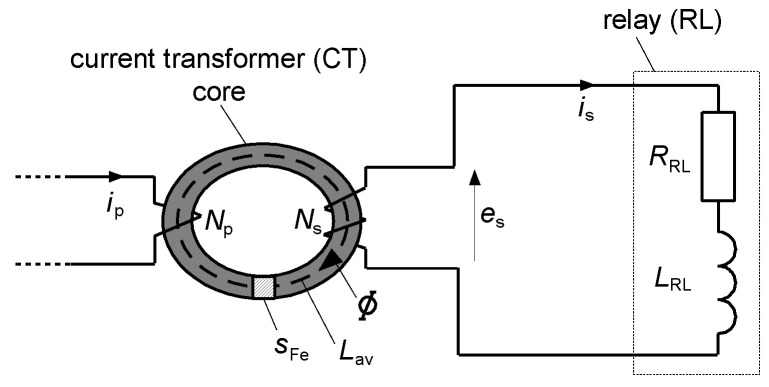
The RCD simplified model for the theoretical analysis. *N*_p_, *N*_s_—the number of turns of the primary and secondary windings of the CT, respectively, *L*_av_—average length of the magnetic flux path in the iron core of the CT, *s*_Fe_—cross-section of the core of the CT, *e*_s_—induced secondary voltage in the CT, *i*_p_—primary current of the CT, *i*_s_—secondary current of the CT, *Φ*—magnetic flux in the iron core of the CT, *L*_RL_, *R*_RL_—parameters (inductance and resistance, respectively) of the relay.

To explain the behavior of RCDs in the presence of the DC component as well as to recommend the parameters of these protective devices to improve their sensitivity against this component, the RCD model, according to the diagram presented in Figure 14, has been prepared and studied.

The RCD operates if the secondary current *i*_s_ has a high enough value to trigger the relay RL. However, this secondary current strictly depends on the secondary voltage *e*_s_ induced in the secondary winding of the CT. Therefore, the production of the voltage *e*_s_ can be assumed as a crucial factor to ensure the operation of the RCD.

Induced secondary voltage *e*_s_ of the current transformer depends on the following parameters (for symbols see Figure 14):

(1) $$e_{s}=N_{s}\frac{d\Phi }{dt}$$

Magnetic flux *Φ* in the iron core of the current transformer is a function of the magnetic induction *B* and cross-section *s*_Fe_ of the core of the current transformer:

*Φ* = *B s*_Fe_ (2)

The flow law has the form:

(3) $$N_{p}\cdot i_{p}-N_{s}\cdot i_{s}=H\cdot L_{\mathrm{av}}$$

where *H* is the magnetic field strength in the iron core.

**Figure 15:**
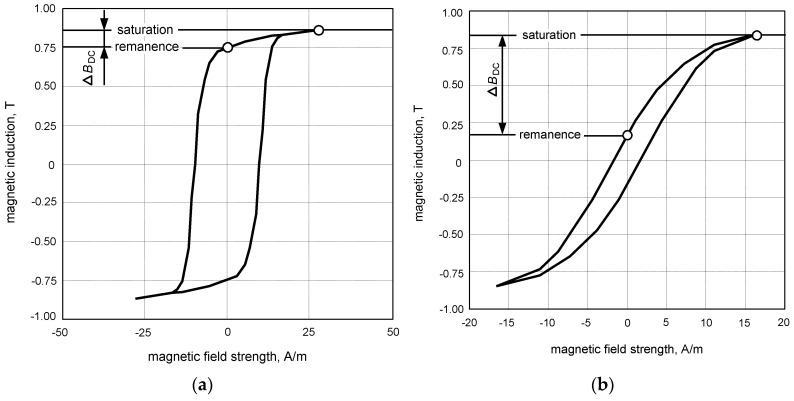
Magnetization characteristics (measured, for 50 Hz) of current transformer cores from RCDs: (**a**) AC-type (here called round hysteresis loop); (**b**) A-type (here called flat hysteresis loop). ∆*B*_DC_—variation of the induction between the remanence point and the saturation point.

The relation between magnetic induction (*B*) and magnetic field strength (*H*) strictly depends on the type of iron core of the CT. Properties of the commonly used magnetic cores are presented in Figure 15.

Unfortunately, when the DC component appears in the current *i*_p_, the secondary voltage *e*_s_ may not be sufficient to produce the secondary current *i*_s_ to initiate tripping of the relay RL. To generate the sufficient induced voltage, not only the DC component content is important, but also the shape of the hysteresis loop of the magnetic core of the CT (Figure 15).

**Figure 16:**
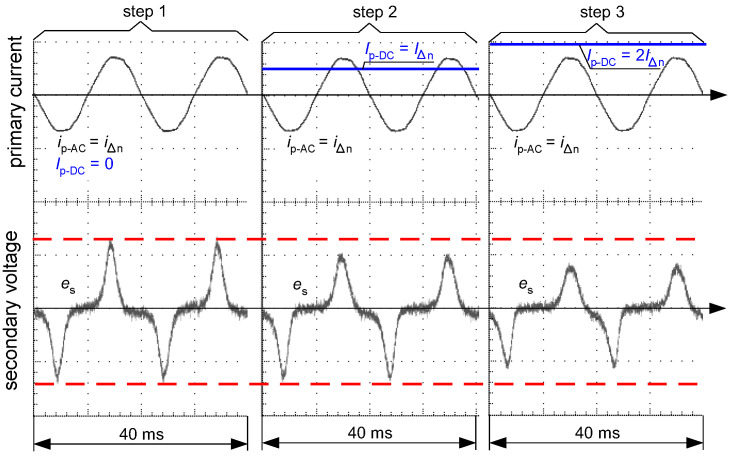
Oscillograms of the induced secondary voltage *e*_s_ in case of the iron core of round hysteresis loop (as in Figure 15a). Primary current *i*_p_ composed of sinusoidal AC waveform (*i*_p-AC_ = *i*_∆n_) and DC component (*I*_p-DC_) of the consecutive values: *I*_p-DC_ = 0 (step 1), *I*_p-DC_ = *I*_∆n_ (step 2), *I*_p-DC_ = 2*I*_∆n_ (step 3).

**Figure 17:**
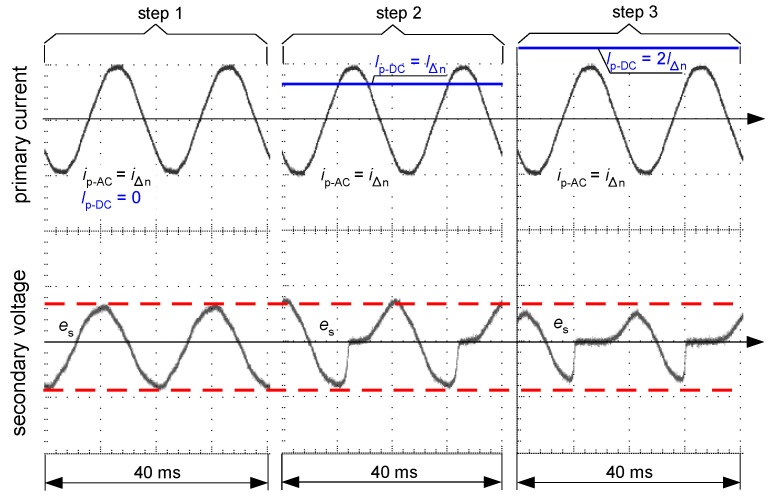
Oscillograms of the induced secondary voltage *e*_s_ in case of the iron core of flat hysteresis loop (as in Figure 15b). Primary current *i*_p_ composed of sinusoidal AC waveform (*i*_p-AC_ = *i*_∆n_) and DC component (*I*_p-DC_) of the consecutive values: *I*_p-DC_ = 0 (step 1), *I*_p-DC_ = *I*_∆n_ (step 2), *I*_p-DC_ = 2*I*_∆n_ (step 3).

Figure 16 and Figure 17 present oscillograms of the secondary voltage waveform *e*_s_ if the primary current *i*_p_ is composed of the following components:

- only sinusoidal of value *I*_∆n_—(step 1);
- sinusoidal of value *I*_∆n_ + smooth DC component of value *I*_∆n_—(step 2);
- sinusoidal of value *I*_∆n_ + smooth DC component of value 2*I*_∆n_—(step 3).

For both types of iron cores of the CT (AC-type—as in Figure 15a, A-type—as in Figure 15b), the induced voltage *e*_s_ decreases if the DC component increases. This makes deterioration of the sensitivity of RCDs, as shown in Figure 6, Figure 7, Figure 8 and Figure 13, but in each of the tested cases the RCDs still tripped.

**Figure 18:**
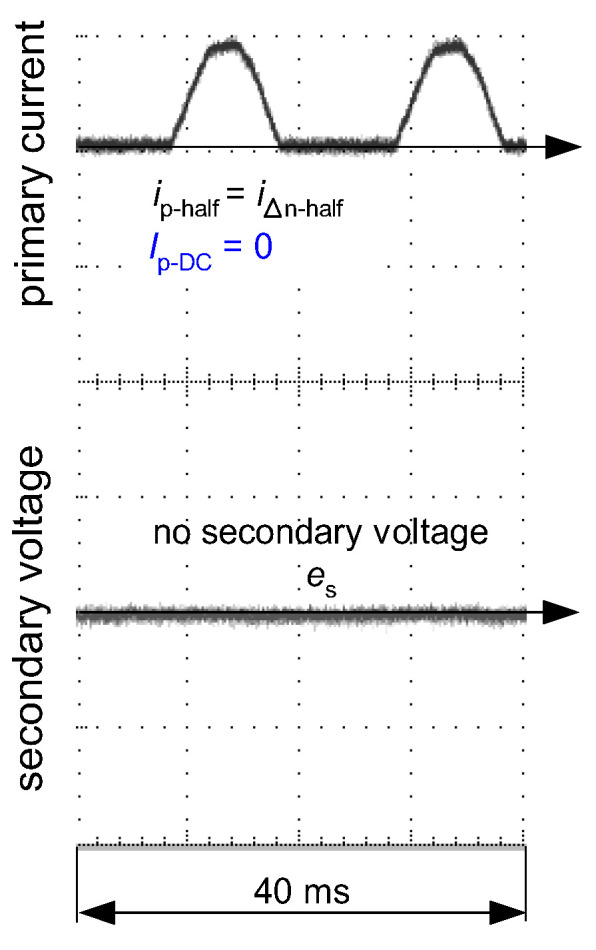
Oscillogram of the induced secondary voltage *e*_s_ in case of the iron core of round hysteresis loop (as in Figure 15a). Primary current *i*_p_ in the form of pulsating DC (half-wave) waveform (*i*_p-half_). No induced secondary voltage *e*_s_.

**Figure 19:**
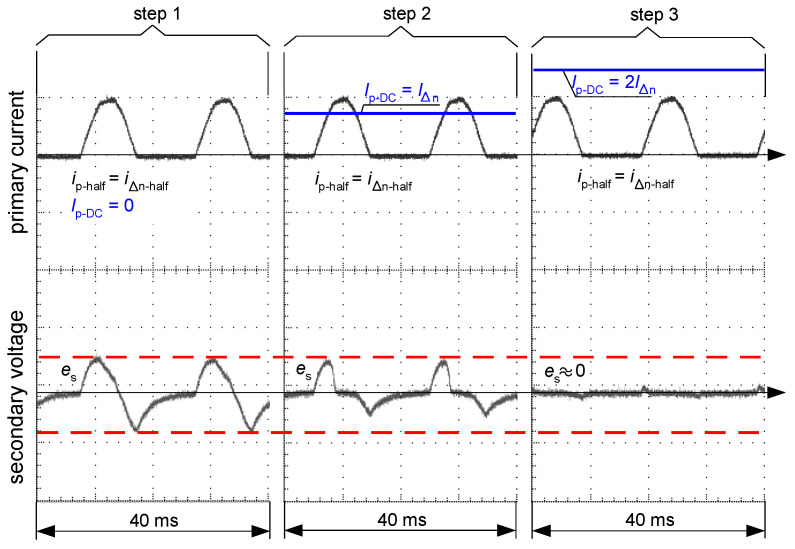
Oscillograms of the induced secondary voltage *e*_s_ in case of the iron core of flat hysteresis loop (as in Figure 15b). Primary current *i*_p_ composed of pulsating DC (half-wave) waveform (*i*_p-half_ = *i*_∆n-half_) and DC component (*I*_p-DC_) of the consecutive values: *I*_p-DC_ = 0 (step 1), *I*_p-DC_ = *I*_∆n_ (step 2), *I*_p-DC_ = 2*I*_∆_ (step 3).

The situation changes when the induced voltage *e*_s_ is analyzed with the following forced primary current *i*_p_ (Figure 18 and Figure 19):

- only pulsating DC (half-wave) of value *I*_∆n-half_—(step 1);
- pulsating DC (half-wave) of value *I*_∆n-half_ + smooth DC component of value *I*_∆n_—(step 2);
- pulsating DC (half-wave) of value *I*_∆n-half_ + smooth DC component of value 2*I*_∆n_—(step 3).

While in the case of the A-type core (flat hysteresis loop), the induced voltage appears even when the DC component is relatively high (Figure 19, step 2), but in the case of the AC-type core (round hysteresis loop), the voltage is not induced even in the absence of the smooth DC component (Figure 18). This is the answer to why the AC-type RCDs responded in the worst way to the testing current, as presented in Figure 13.

Thus, a key element in detecting waveforms with a DC component is the magnetization characteristics of the iron core of the CT. One should strive for the production and use of cores with the lowest possible value of remanence induction *B*_rem_, so that, with a high share of the DC component, the highest change in the magnetic induction can be achieved—compare ∆*B*_DC_ in Figure 15a vs. Figure 15b.

**Figure 20:**
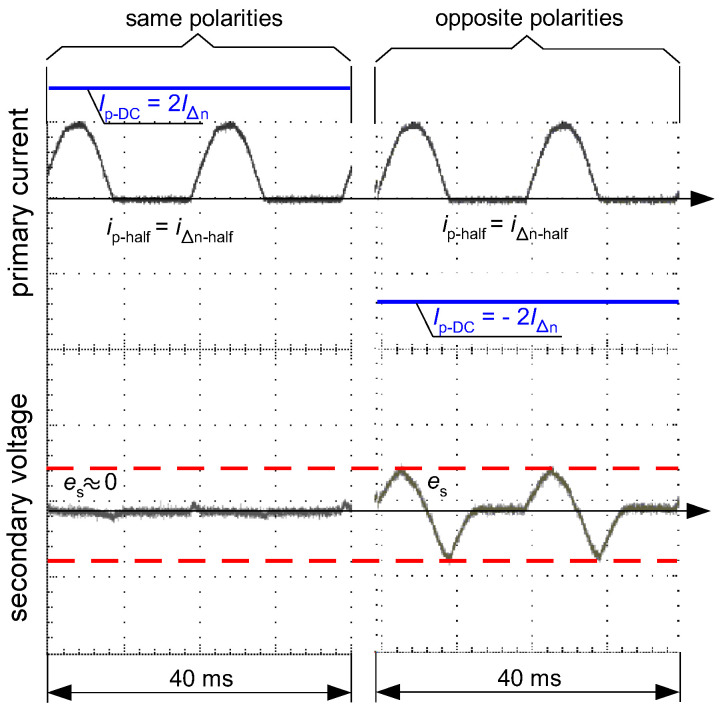
Oscillograms of the induced secondary voltage *e*_s_ in case of the iron core of flat hysteresis loop (as in Figure 15b). Primary current *i*_p_ composed of pulsating DC (half-wave) waveform (*i*_p-half_ = *i*_∆n-half_) and DC component (*I*_p-DC_) of the value: *I*_p-DC_ = 2*I*_∆n_. Case 1 (**left**)—same polarities of the components; Case 2 (**right**)—opposite polarities of the components.

When the half-wave component has the opposite polarity to the smooth DC component, the operating conditions for the RCD could be favorable, as shown in Figure 11. This phenomenon is explained in Figure 20. The opposite polarity of these components causes the resultant waveform to become bidirectional (or with high pulsation) and therefore a relatively high level of the voltage *e*_s_ is induced in the secondary winding of the CT (Figure 20, case 2—right). Such a case may give an unexpectedly positive response of the RCD, i.e., its tripping, as reflected in the aforementioned Figure 11.

### 3.4. The Suddenly Applied Smooth Direct Current

In this type of RCDs testing (TEST 3), only a pure direct current is forced. After adjusting parameters of the testing circuit (generator G_DC_ and rheostat *R*_DC_ in Figure 5), one of the following values of the current starts to flow 15, 20, 30, 60, 90, 150, 300, or 600 mA. During this test, it has been verified whether RCDs reacted to the suddenly applied direct current and disconnected the supply. They were tested:

- nine A-type RCDs of a rated residual operating current *I*_∆n_ = 30 mA;
- two F-type RCDs of a rated residual operating current *I*_∆n_ = 30 mA.

**Figure 21:**
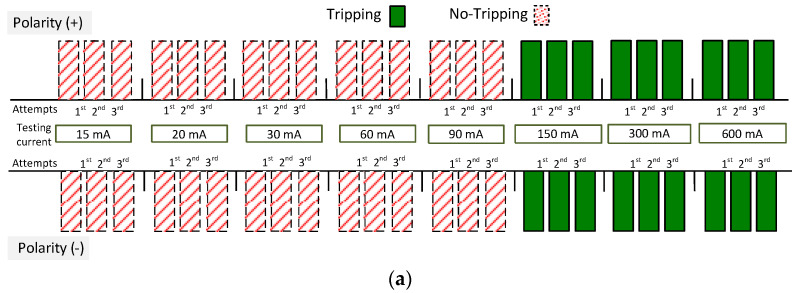

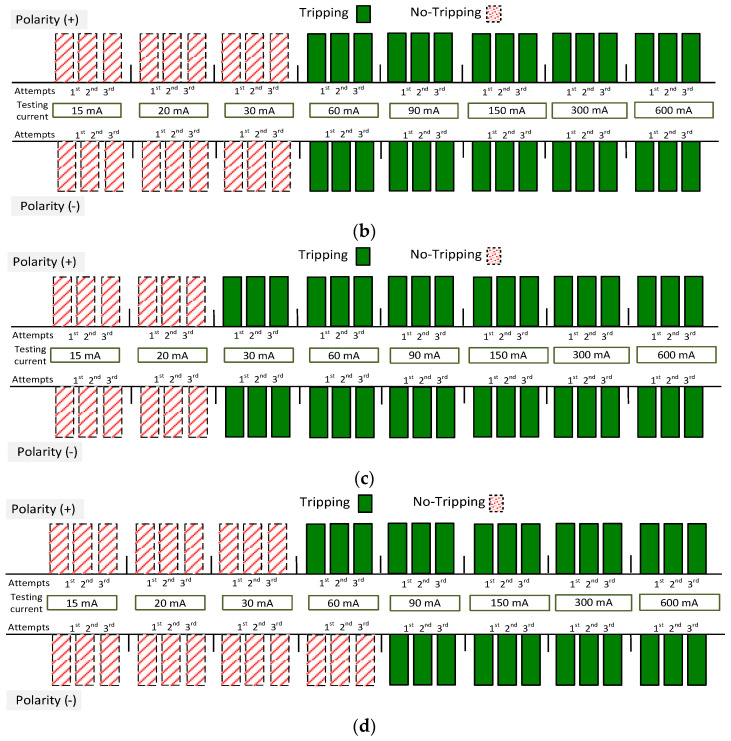
Tripping of the 30 mA A-type RCDs for a pure direct current (suddenly applied) of values 15, 20, 30, 60, 90, 150, 300 and 600 mA. Tested RCDs: (**a**) (RCD30_6); (**b**) (RCD30_7); (**c**) (RCD30_1); (**d**) (RCD30_8).

**Figure 22:**
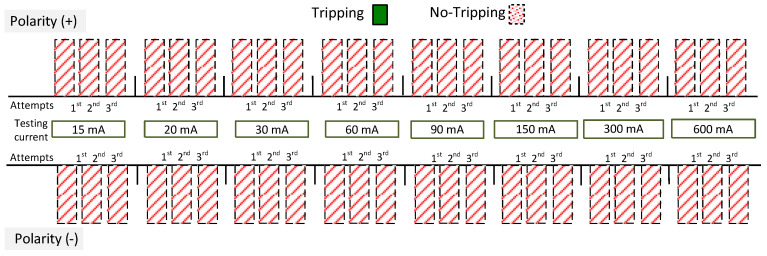
Tripping of the two 30 mA F-type RCDs: (RCD30_4) and (RCD30_5) (the same responses for these two RCDs) for a pure direct current (suddenly applied) of values 15, 20, 30, 60, 90, 150, 300 and 600 mA.

Selected results (the most interesting because they are varied) for four A-type RCDs are depicted in Figure 21; both F-type RCDs are depicted in Figure 22. The results in these figures are expressed in the form of bars. If the reaction of the RCD occurred, the annotation “Tripping” was performed—otherwise “No-Tripping” was noted. The testing of RCDs has been performed for both polarities of the direct current: (+) and (−). Moreover, for every polarity, each value of the current was forced three times (in Figure 21 and Figure 22 it is marked by bars “1st, 2nd, 3rd”) with an interval of around 20–30 s between consecutive attempts.

The results obtained for RCD30_6 (Figure 21a) inform that this RCD reacted to the direct current values of 150, 300, and 600 mA (each attempt) regardless of polarity. A better response to a suddenly applied direct current was characterized by RCD30_7 (Figure 21b)—it reacted to direct currents of 60 mA and above. Very interesting results were obtained for RCD30_1 (Figure 21c)—this RCD reacted to direct currents starting from 30 mA, which is, after all, its rated residual operating current. Such a behavior is very favorable—it is usually required and obtained for a sinusoidal current of a rated frequency. A slightly different behavior was noted in the case of RCD30_8 (Figure 21d). Here, the polarity of the direct current influences the tripping level. For positive polarity, it operated within the range of 60–600 mA, and for negative polarity, within the range of 90–600 mA. Nevertheless, its reaction to suddenly applied direct current can be described as favorable.

If one assesses the results obtained for F-type RCDs (Figure 22—both RCDs did not react at all), it could be said that their properties in relation to the detection of direct currents suddenly applied are worse than in the case of A-type RCDs. This seems to be in contradiction with normative requirements and results of TEST 1 (compare Figure 6a vs. Figure 6b). The reason lies, paradoxically, just in the normative requirements.

According to the standard [37], F-type RCDs are required to withstand inrush residual currents having a duration of up to 10 ms. This requirement allows to avoid the unwanted tripping of F-type RCDs in circuits with electromagnetic filters connected to the protective conductor PE (earthing system). Consequently, F-type RCDs can surprisingly well detect slowly increased residual currents containing a large DC component (see Figure 6b) but will not respond to a suddenly applied direct current. One should be aware that the sudden application of direct current, even of relatively small value, causes its rapid increase (a high gradient/rise rate), which is interpreted by F-type RCDs as an inrush current (therefore they do not react).

**Figure 23:**
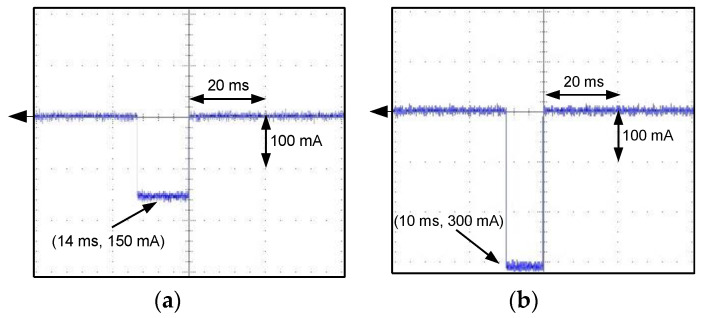
Oscillograms of the DC current flow during the tripping test of the A-type RCD (RCD30_6). Suddenly applied DC current of the following values: (**a**) 150 mA; (**b**) 300 mA.

**Figure 24:**
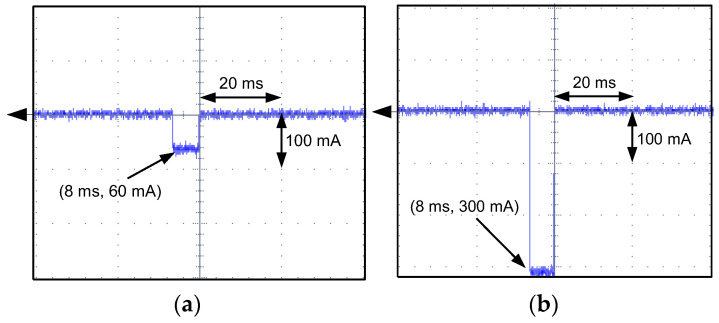
Oscillograms of the DC current flow during the tripping test of the A-type RCD (RCD30_7). Suddenly applied DC current of the following values: (**a**) 60 mA; (**b**) 300 mA.

**Figure 25:**
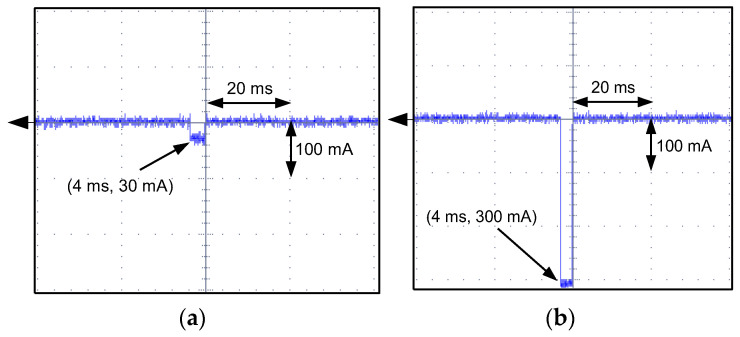
Oscillograms of the DC current flow during the tripping test of the A-type RCD (RCD30_1). Suddenly applied DC current of the following values: (**a**) 30 mA; (**b**) 300 mA.

**Figure 26:**
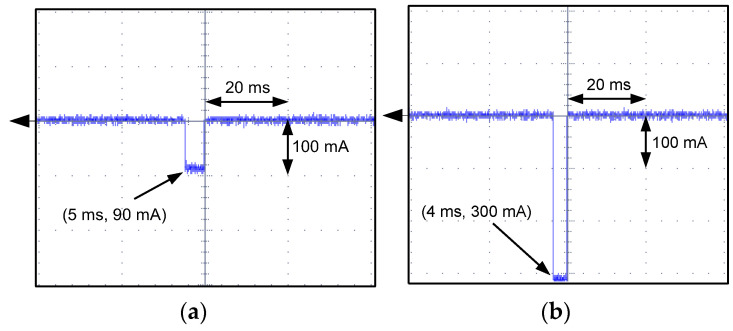
Oscillograms of the DC current flow during the tripping test of the A-type RCD (RCD30_8). Suddenly applied DC current of the following values: (**a**) 90 mA; (**b**) 300 mA.

For A-type RCDs with tripping results presented in Figure 21, the oscillograms of the residual current during the RCDs’ operation have been recorded (selected, example values; Figure 23, Figure 24, Figure 25 and Figure 26). These oscillograms clearly show a very fast increase in the current after switching on the laboratory testing circuit. The RCDs without any delay in tripping (as tested A-type RCDs; they have no designed delay in response) have favorable conditions to disconnect the supply. The very fast increase in the current may induce a sufficient voltage in the secondary winding of the CT (*e*_s_ voltage in Figure 4), and then the appropriate value of the secondary current *i*_s_ initiates tripping of the relay RL—hence, also the RCD. Analysis of the oscillograms for various values of predetermined residual direct current enables to conclude that this current has a form of a rectangular half-wave and its flow time is very short (up to 14 ms). Moreover, this time depends to a small extent on the value of the current. This is the effect of a very high rise rate of the wave just after the circuit is switched on.

**Figure 27:**
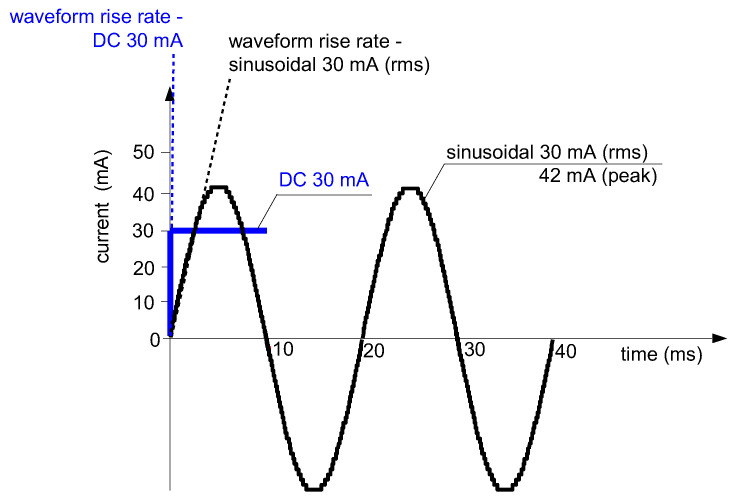
The rise rates of the sinusoidal 50 Hz AC (30 mA rms) and DC (30 mA) waveforms.

If comparing a sinusoidal waveform vs. a rectangular waveform (Figure 27), it is easy to conclude that the latter may give higher induced voltage *e*_s_, described by (1), than the sinusoidal waveform. This voltage *e*_s_ is the function of the residual (primary) current rise rate d*i*_∆_/d*t* (d*i*_p_/d*t*). Such a property may give an unexpectedly positive reaction to a DC component that is suddenly applied.

Thus, as the investigation has shown, the detection of suddenly applied direct current by no-delayed A-type RCDs is relatively easy and their tripping for direct current of value equal to even *I*_∆n_ is probable, although they are not normatively designed to detect such a waveform.

**Figure 28:**
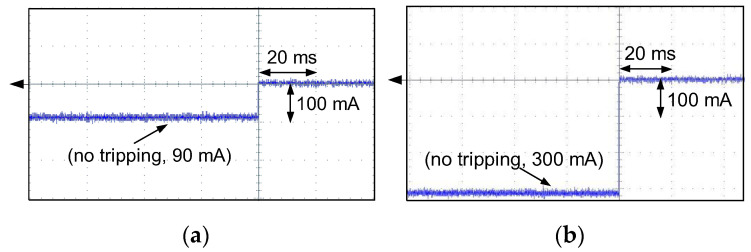
Oscillograms of the DC current flow during NO tripping of the F-type RCD (RCD30_5). Suddenly applied DC current of the following values: (**a**) 90 mA; (**b**) 300 mA.

The respective oscillograms recorded for F-type RCD30_5 (Figure 28) confirmed that it has no reaction to the testing current. After the test circuit is switched on, a direct current of a steady value flows in it (the RCD is still in the closed position).

## 4. Conclusions

RCDs are widely used in low-voltage electrical installations. Undoubtedly, they contribute to the improvement of electrical safety, but their selection must be considered carefully. The main issue determining the correctness of the earth fault/residual current detection in circuits with power electronic converters is the selection of RCDs due to their ability to react to specific waveform shapes of the current. In PV installations and in EV charging installations, one should take into account the possibility of the appearance of a DC component in the residual current.

As shown by the results of above-presented laboratory tests, A-type and F-type RCDs are able to detect slowly rising residual current with a very high content of the DC component. Many of the tested RCDs met the requirements of the relevant standards to a large excess—e.g., for A-type RCDs the max normative value of the smooth DC component is equal to only 6 mA (in the composite waveform: half-wave + smooth DC component) but some of them tripped properly for the DC component up to a maximum forced value of 150 mA. Therefore, it seems reasonable for RCDs’ manufacturers to include, along with their products, the information about the real ability of RCDs to detect residual currents with a DC component. It is especially important for proper use of RCDs, because devices with higher rated residual operating currents (*I*_∆n_ = 100 or 300 mA) may operate, as it has been experimentally verified, within the range (0.5–1.0)*I*_∆n_, even if a DC component is many times higher than the normative value (6 mA or 10 mA) specified in current international standards. Another important factor is the type of iron core of the current transformer installed inside the RCD. It is highly recommended that RCD manufacturers select magnetic cores with a particularly low remanence magnetic induction. Considering that in modern installations one can expect a large share of the DC component in the earth fault current, such a core (low remanence, flat hysteresis loop) will allow the detection of a current with a very high DC component.

With regard to the response of RCDs to suddenly applied direct current, there is a very important remark. The responses of most of the tested RCDs to such a current were very good, provided that the RCD was of the no-delayed type. RCDs that, according to relevant standards, have a guaranteed delay in operation, cannot react to a suddenly applied direct current. This unfavorable behavior applies to F-type RCDs (a normative delay is equal to 10 ms), which have very good properties for a slowly rising residual current containing a DC component. Therefore, it is strongly postulated that two types of F-type RCDs should be introduced into the standardization: the 1st type—a no-delayed RCD; the 2nd type—a short-delayed RCD (as it is now). It is expected then that F-type RCDs operating without any delay could detect the suddenly applied direct current very well, which is very important for the effective protection against electric shock in installations containing direct current sources. This is also an economic advantage as in some cases, F-type RCDs may be installed instead of B-type.

## Figures and Tables

**Table 1:** Types of RCDs and sample normative waveforms *i*_T_ with DC component (or direct current of low pulsation) for RCDs testing.

RCD Type	Ability to Detect	View of the Example Waveforms
AC	– sinusoidal (50/60 Hz) residual currents	no tests under DC component
A	– sinusoidal (50/60 Hz) residual currents; – pulsating direct residual currents	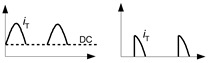 DC component max 6 mA
F	– sinusoidal (50/60 Hz) residual currents; – pulsating direct residual currents; – mixed-frequency residual currents generated by control equipment supplied from a single-phase	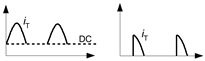 DC component max 10 mA
B	– sinusoidal residual currents (frequency up to 1000 Hz); – pulsating direct residual currents; – sinusoidal currents superimposed by a smooth direct component; – smooth direct residual currents; – mixed-frequency residual currents	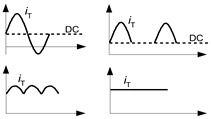

## Data Availability

Data is contained within the article.
